# Resident-as-Teacher Curriculum: An Evidence-based Guide to Best Practices from the Council of Residency Directors in Emergency Medicine

**DOI:** 10.5811/westjem.41493

**Published:** 2025-09-24

**Authors:** Jaime Jordan, Michael Gottlieb, Molly Estes, Melissa E. Parsons, Katja Goldflam, Andrew Grock, Brit J. Long, Sree Natesan

**Affiliations:** * David Geffen School of Medicine at University of California Los Angeles, Department of Emergency Medicine, Los Angeles, California; † Oregon Health & Science University, Department of Emergency Medicine, Portland, Oregon; ‡ Rush University Medical Center, Department of Emergency Medicine, Chicago, Illinois; § Northwestern University, Department of Emergency Medicine, Chicago, Illinois; ¶ Brooke Army Medical Center, Department of Emergency Medicine, San Antonio, Texas; || University of Florida College of Medicine, Department of Emergency Medicine, Jacksonville, Florida; # Yale School of Medicine, Department of Emergency Medicine, New Haven, Connecticut; ** Duke University, Division of Emergency Medicine, Durham, North Carolina

## Abstract

Improving resident teaching skills is an expectation of training. Despite the recognized importance of resident-as-teacher (RaT) curricula, variability indicates the need for evidence-based guidelines to inform best practices. This paper outlines expert guidelines for the development, implementation, and evaluation of RaT curricula from the members of the Council of Residency Directors in Emergency Medicine Best Practices Subcommittee, based on a critical review of the literature. It is important to perform a needs assessment prior to creating and implementing a RaT curriculum. The RaT curricula should include instruction on adult learning theory, feedback, and classroom and bedside teaching techniques. Outcomes of RaT curricula should be assessed using multiple sources including direct observation and incorporate both knowledge and skill retention, as well as acquisition.

## BACKGROUND

Training future physicians to be teachers is an important curricular component of residency programs and supported by the Accreditation Council for Graduate Medical Education (ACGME), which states that residents are expected to participate in the education of patients, families, students, residents, and other health professionals and should be encouraged to teach using a scholarly approach.^1^ Resident-as-teacher (RaT) curricula hold the potential to provide numerous benefits to residents, medical students, and patients by enhancing teaching skills that allow for transfer of knowledge.^2^–^17^ Benefits of RaT programs across medical specialties include improved teaching skills, self-reflection, self-efficacy in teaching, and improved educational outcomes for both residents and their learners, as well as better outcomes for patient care.^2^–^17^

Despite recommendations to provide this training in residency and a substantial body of literature on the topic, there is no standard approach to RaT curricula.^1^ This deficit can lead to variability in education skill development for resident trainees. It also leaves education leaders uncertain about how to best provide this important training in their programs. While a few prior reviews have sought to address this topic, they include only a small number of papers, are narrow in scope (focusing on the benefits and effectiveness of RaT curricula rather than how to best deliver this type of instruction), and may be outdated and not reflect the current literature available.^6^,^9^,^13^,^17^ Therefore, a critical need exists to develop best practices and evidence-based guidelines to optimize RaT curricular content, implementation, and evaluation in graduate medical education training programs.

Based on the best available evidence through a critical review of the literature, we offer expert guidelines on RaT curricular content, implementation, and evaluation from members of the Council of Residency Directors in Emergency Medicine (CORD) Best Practices Subcommittee. This paper provides readers with recommendations on the content, educational strategies, curricular implementation, and program evaluation for RaT curricula.

## CRITICAL APPRAISAL

This is the 11^th^ paper in a series of evidence-based best practice reviews from the CORD Best Practices Subcommittee.^18^–^27^ The author group consists of expert emergency medicine (EM) educators and education researchers with experience in residency program education and leadership. We conducted a literature search in conjunction with a medical librarian using MEDLINE with a combination of Medical Subject Heading terms and keywords focused on RaT curricula searching for papers published from inception to December 31, 2023 (Supplemental Appendix 1). We also reviewed the bibliographies of all included papers. Two authors (JJ and SN) independently screened and included papers that addressed RaT curricula development, implementation and evaluation. We excluded papers that were not related to RaT curricula development, implementation, or evaluation. We also excluded papers that were not in English, were abstracts only, or did not have full text available. Papers were included based on agreement of the two screeners. The two screeners resolved discrepancies through in-depth discussion and negotiated consensus.

The search yielded 1,486 papers, of which 89 were deemed to be directly relevant to this review (Supplemental Appendix 2). The author group derived their best practice recommendations based on the literature review and discussion among the expert author group. The level and grade of evidence were provided for each best practice statement implementing the Oxford Center for Evidence-Based Medicine criteria (Tables 1 and 2).^28^ When supporting data were not available, recommendations were made based upon the authors’ combined experience and consensus opinion. Prior to submission, the manuscript was reviewed by the CORD Best Practices Subcommittee and posted to the CORD website for two weeks for peer review by the entire CORD medical education community. Upon completion of the review period, there was general agreement, and no substantial changes to the guideline were recommended.

**Population Health Research Capsule**

What do we already know about this issue?

*Resident-as-teacher (RaT) curricula are an important part of residency training and have many potential benefits*.

What was the research question?

*What are best practices for RaT curricular content, implementation, and evaluation in graduate medical education training programs?*

What was the major finding of the study?

*This paper offers expert recommendations for best practices on RaT curricular content, implementation, and evaluation*.

How does this improve population health?

*Improving teaching skills ultimately leads to better education outcomes for residents and better care of their patients*.

## RESIDENT-AS-TEACHER CURRICULAR CONTENT AND EDUCATIONAL STRATEGIES

Of the reviewed papers, few included a formal needs assessment beyond a review of the literature. Residents’ responsibility to teach students, other residents, and other staff is well recognized, as is the need to provide training to prepare residents for their roles as teachers.^29^ Reasons for implementing RaT curricula include the following: to teach a skill important to the resident role; meet residents’ desire for formal training in education; address regulatory requirements; and prepare trainees for future career roles.^1^,^30^ General curricular goals included improving resident formal and informal teaching skills in both classroom and clinical settings and increasing resident confidence in teaching skills.^29^,^31^ The RaT curricula reviewed contain diverse components. The topics most consistently included in RaT curricula were adult learning theory, creating a positive learning environment and setting objectives, clinical or bedside teaching techniques, classroom teaching techniques, and how to give feedback.^10^–^12^,^14^,^15^,^29^–^60^

Adult learning theory—which describes how adults learn best when material is problem-centered, relevant to their work, and when they are involved in the planning and evaluation of their instruction—was a major component of RaT curricula, both as a framework for the curricular development and a topic of instruction for learners.^10^,^29^,^31^–^42^ Adult-learning theory was often considered in how RaT curricula was applied.^34^,^35^,^61^ For example, RaT leaders factored this in for determining the length, frequency, and formatting of these educational sessions within the curricula. 34,35,61

Many curricula also include adult learning principles as part of their educational content.^11^,^29^, ^33^,^35^–^42^,^49^,^56^ Berger et al provided a primer for anesthesiology residents about adult learning principles by having the learners discuss effective and ineffective teaching moments that they remembered in their education.^11^ They also had learners review literature on adult learning principles and watch a video demonstration.^11^ Similarly, Chee et al had residents identify effective and ineffective teaching strategies observed in video clips to better understand adult learning theory.^35^ Choski et al had learners review two papers on adult learning theory to better understand adult education principles.^36^ Another group used formal lectures on adult learning theory followed by debriefing.^29^ Tang Girdwood et al revised a previous curriculum by removing the PowerPoint lecture on adult learning theory and instead having residents teach the principles of adult learning theory to one another with a faculty facilitator present.^42^

Many RaT curricula sought to teach residents how to set the stage for learning.^11^,^12^,^15^, ^31^,^32^,^34^–^36^,^38^,^43^–^49^ Curricular content included how to create a positive learning environment and recognize behaviors that can lead to an environment of harassment or learner mistreatment.^12^,^31^,^35^,^43^,^44^ Understanding how to set goals and expectations with learners to facilitate knowledge and skill acquisition was also an important topic included in RaT curricula.^11^,^15^,^31^,^32^,^34^,^36^,^38^,^44^–^49^

Clinical or bedside teaching techniques and tools was another commonly included topic in RaT curricula.^10^,^14^,^15^,^29^–^31^,^33^,^37^–^42^,^44^,^46^–^48^,^50^–^55^ One survey study in EM found that 84% of programs reported bedside teaching to be a major focus of their educational curriculum.^32^ One of the most frequently included teaching tools was the One-Minute Preceptor.^31^,^32^,^37^,^44^,^47^,^51^,^52^,^54^,^57^,^62^ Ahn et al found that 45% of RaT programs in a single specialty incorporated training on the One-Minute Preceptor.^32^ In another example, curricula learners were asked to describe the elements of this model, apply the model to a simulated learner’s patient presentation, and use the model to assess the learner’s knowledge level and identify educational points.^31^ Content specific to procedural teaching was included in many curricula. 5,10,11,15, 29,32,33,35–37,41,46,53,55,57,59

In addition to the clinical setting, many RaT curricula also seek to prepare residents for teaching in the classroom by including content on didactic, small group, and case-based instruction.^11^,^15^,^31^,^32^,^38^,^41^,^42^, ^45^,^46^,^48^,^50^,^53^,^54^,^56^–^58^ While these content areas were often listed as topics or titles of educational sessions included in curricula, there was little additional description in the included studies as to what these content areas were comprised of. Many curricula also included content on the use of simulation in education.^14^,^32^,^42^,^53^,^55^,^57^,^63^

Feedback was also consistently included in RaT curricula.^8^,^10^–^12^,^14^,^15^,^29^,^31^–^38^,^40^–^44^,^46^–^48^,^50^,^52^,^53^,^55^,^59^,^60^ One study found that 96% of EM residency programs that had RaT curricula included feedback as a major focus.^32^ Specific content areas related to feedback included techniques and components of effective feedback, optimizing the environment for feedback, and how to receive feedback.^33^ Curricula often included interactive activities, during which the learners could practice feedback interactions via role-play and debrief with the other learners.^30^,^33^

Other RaT curricular content included education to augment teaching such as communication skills, professionalism, and how to deal with difficult learning situations.^32^,^46^,^50^,^57^,^58^ Some curricula also included content that could help prepare residents as education professionals such as mentorship and role modeling, curricular design, time management, and learner assessment.^15^,^32^,^40^,^46^,^57^,^60^,^64^ We provide a summary of RaT curricular content and educational strategies in Tables 3 and 4.

**Table:** 

**Best Practices Recommendations**
**Resident-as-teacher curricula should include the following:** Teaching techniques applicable to both classroom and bedside settings (Level 1, Grade A). Effective feedback techniques that educators can use to provide feedback to learners (Level 1, Grade B). Adult learning theory as part of the framework of the curriculum and its delivery, as well as an educational component of the curriculum. (Level 2, Grade B).

## RESIDENT-AS-TEACHER CURRICULAR LOGISTICS AND IMPLEMENTATION

Timing, duration, and frequency of interventions varied greatly among studies and specialties, with no overarching consensus on ideal approaches. The most common approach included single interventions, usually early in intern year or during residency orientation, with most one-day curricula ranging from 4–8 hours.^44^, ^47^, ^59^, ^60^, ^62^, ^65^–^67^ According to a landmark paper published by Morrison et al in 2004, the average total time for a RaT curriculum was 11 hours, with their institutional published curriculum lasting for 13 total cumulative hours of longitudinal instruction.^68^, ^69^ Some longitudinal curricula had longer durations including those that spanned the entire length of resident training.^12^, ^29^, ^31^, ^38^, ^42^, ^49^, ^54^, ^55^

Staffing of the educational sessions was largely by general residency faculty who participated in didactics, mentorship, or evaluations of resident teaching.^56^, ^58^, ^59^, ^62^, ^69^, ^70^ Sometimes faculty with additional training or specialization in education led or designed the curricula, which included “educational experts,” designated education faculty, and education fellows.^12^, ^15^, ^70^ Additionally, residents themselves often contributed, including chief residents and teach-the-teacher models.^47^, ^58^

Several barriers were identified in the implementation of RaT curricula, with the most frequently mentioned being the balance of workload on faculty and residents.^71^, ^48^, ^58^ Both the total time required for participation and instruction as well as real-time balancing responsibilities of patient care with teaching while working clinically were noted.^37^, ^57^, ^72^, ^73^ Additionally, many residents felt it was challenging to teach topics that they themselves still did not feel quite familiar with, even for the sake of experiential learning.^37^, ^43^, ^57^ Lastly, despite ACGME supportive program requirements, some program directors felt that RaT curricula were not a priority among other competing educational demands.^1^, ^58^,^74^

A needs assessment before creating and implementing a RaT curriculum can help confirm interest, elucidate clear, specific program goals for participants, and secure buy-in from faculty and leadership.^37^, ^56^, ^58^, ^75^ Buy-in from residents was less challenging, with many residents confirming that they lacked self-confidence in their own teaching abilities, wanted mentorship in this area, and were willing to spend time to gain this experience.^5^, ^59^, ^76^ Medical students, who along with junior residents, were frequently the recipients of the outcomes of RaT, identified residents as more approachable than faculty and appreciated near-peer teaching.^73^, ^77^–^79^

Administration of RaT curricula may be challenging due to the resources required for successful implementation. This includes the number of faculty needed and time for residents to participate in curricular sessions, as well as time to learn and practice these skills while working clinically.^58^,^70^ Through an online survey of 47 residency programs and iterative expert consensus building, McKeon et al proposed the following key components to a successful RaT curriculum: required trainee participation; evaluations and feedback of resident teaching; recognition of excellence through teaching awards; and faculty teaching evaluations linked to annual faculty review but not to salary or promotion.^80^ Finally, a RaT curriculum should be iteratively refined to ensure optimization of its content.^42^

**Table:** 

**Best Practices Recommendations:** General residency faculty can teach, provide mentorship, and evaluate participants in RaT curricula (Level 2a, Grade B). Perform a needs assessment prior to implementing a RaT curriculum (Level 3a, Grade B). Identify and address barriers such as time limitations for residents and faculty when implementing a RaT curriculum (Level 4, Grade C).

*RaT*, resident as teacher.

## RESIDENT-AS-TEACHER CURRICULAR OUTCOMES/EVALUATION

When evaluating a RaT program, it is critical to use a robust model, accounting for various inputs, outputs, and outcomes. Examples of relevant program evaluation frameworks include the Kirkpatrick framework, the Logic Model, and CIPP (Context, Input, Process, Products).^81^,^82^ Despite this, most studies did not explicitly state the program evaluation framework they used.

While many studies included only a single or limited number of outcome measures individually, when assessed as a whole, there were a wide range of potential outcomes assessed (Table 5). The most common form of learner assessment was self-surveys of perceived effectiveness after a RaT program.^4^–^7^, ^9^–^12^, ^14^, ^31^, ^33^, ^35^, ^39^, ^42^, ^44^, ^48^, ^49^, ^51^, ^54^–^56^, ^58^, ^61^, ^62^, ^65^, ^66^, ^71^, ^74^, ^83^–^97^ A few studies also conducted delayed self-assessments at 3–12 months following RaT course completion.^11^,^44^, ^51^ One study assessed differences in attitude toward teaching after the course, while others performed knowledge assessment tests.^36^, ^43^, ^44^ Another study assessed actual use of the skills in subsequent teaching.^52^

Skill assessments were performed using either direct observation or structured assessments in a simulated environment. Several studies directly observed resident teaching, while others video-recorded resident teaching for delayed assessments.^6^, ^49^, ^71^, ^72^, ^74^, ^87^ Other measures included end-of-shift teaching evaluations completed by faculty.^56^, ^58^, ^74^ The most common assessment, using simulation, was the Objective Structured Teaching Exercise (OSTE).^6^, ^9^, ^36^, ^38^, ^41^, ^46^, ^49^, ^54^, ^63^, ^71^, ^74^, ^84^, ^87^, ^92^, ^96^, ^98^ The OSTEs were incompletely reported; they often ranged from 6–8 stations and were 2–4 hours in length. One study used the Debriefing Assessment for Simulation in Healthcare (DASH) instrument instead of the OSTE.^14^ Another assessed both initial and delayed OSTE as part of a randomized trial.^75^

Additional measures were obtained via learners (eg, students, junior residents). Learner assessments used a variety of measures of teaching effectiveness, although most had limited validity evidence.^4^, ^6^, ^9^, ^10^, ^14^, ^39^, ^47^, ^48^, ^62^, ^66^, ^71^, ^74^, ^84^–^89^, ^97^, ^99^, ^100^ One study used the Stanford Faculty Development Program—a 25-item tool assessing learning climate, control of teaching sessions, communicating goals, promoting understanding and retention, evaluation, feedback, and promoting self-directed learning.^47^ Another study evaluated the effect of the intervention by comparing course/rotation evaluations from students.^48^

One study focused on the feasibility to inform broader implementation.^38^ A few other select studies assessed organizational changes and broader outcomes. Two studies found that the RaT program led to substantive changes, which resulted in residency programs converting to this model going forward.^60^,^101^ Others assessed downstream effects on student learning by comparing student Objective Structured Clinical Examinations (OSCE) or Objective Structured Assessments of Technical Skills (OSATS) between those taught by residents completing the RaT program vs those who did not.^63^, ^102^

**Table:** 

**Best Practices Recommendations:** RaT outcomes should be assessed using multiple sources of data (Level 1b, Grade B). Use OSTE or direct observation to directly assess RaT outcomes (Level 1b, Grade B). Incorporate delayed assessment for skill retention (Level 1b, Grade B). Use higher level outcome assessments, such as learner evaluations or assessments (Level 3b, Grade B).

*RaT*, resident as teacher; *OSTE*, Observed Structured Teaching Evaluation.

## LIMITATIONS

Although we performed a comprehensive search guided by a medical librarian in conjunction with a bibliographic review and expert consultation to augment content when needed, we used a single search engine, and it is possible that we may have missed some pertinent papers. In instances where evidence in the form of high-quality data was limited or lacking, we relied upon expert opinion and group consensus for the best practice recommendations. Finally, in areas where evidence was not available, we used the consensus from the expertise of our authorship group. While our author group possesses experience in research and scholarship in both RaT curricula and medical education, there was a potential for bias to have been introduced during this process. Therefore, we also sought peer review from the CORD Best Practices Subcommittee and posted it online for open review feedback by the CORD community.

## CONCLUSION

Resident-as-teacher curricula are a vital component of graduate medical education training programs. This paper provides guidance on best practices for developing, implementing, and evaluating RaT curricula.

## Supplementary Information





## Figures and Tables

**Table 1:** Oxford Centre for Evidence-Based Medicine levels of evidence.^28^

Level of Evidence	Definition
1a	Systematic review of homogenous RCTs
1b	Individual RCT
2a	Systematic review of homogenous cohort studies
2b	Individual cohort study or a low-quality RCT*
3a	Systematic review of homogenous case-control studies
3b	Individual case-control study**
4	Case series/Qualitative studies or low-quality cohort or case-control study***
5	Expert/consensus opinion

* Defined as <80% follow up;

** includes survey studies and cross-sectional studies;

*** defined as studies without clearly defined study groups.

*RCT*, randomized controlled trial.

**Table 2:** Oxford Centre for Evidence-Based Medicine grades of recommendation.^28^

Grade of Evidence	Definition
A	Consistent Level 1 studies
B	Consistent Level 2 or 3 studies or extrapolations* from Level 1 studies
C	Level 4 studies or extrapolations* from Level 2 or 3 studies
D	Level 5 evidence, or troublingly inconsistent or inconclusive studies of any level

* “Extrapolations” refers to data being used in a situation that has potentially clinically important differences than the original study situation.

**Table 3:** Summary of content in resident-as-teacher curricula.

Curricular Content	Number of Papers	References
Adult learning theory	14	8, 10, 29, 31, 33–42
Assessment of learners	1	60
Case-based instruction	7	8, 11, 42, 46, 54, 57, 58
Clinical/bedside instruction	23	10, 14, 15, 29, 30, 31, 33, 37–42, 44, 46–48, 50–55
Communication skills	4	8, 46, 57, 58
Creating a positive learning environment	5	12, 31, 35, 43, 44
Curriculum design	2	8, 46
Didactic instruction	10	11, 15, 31, 38, 42, 45, 48, 50, 53, 54
Difficult learning situations	2	8, 50
Feedback	28	8, 10–12, 14, 15, 29, 31, 33–38, 40–44, 46–48, 50, 52, 53, 55, 59, 60
Mentorship	2	8, 40
Procedural instruction	16	5, 8, 10, 11, 15, 29, 33, 35–37, 41, 46, 53, 55, 57, 59
Professionalism and role modeling	5	8, 15, 46, 57, 64
Setting goals and expectations	13	8, 11, 15, 31, 34, 36, 38, 44–49
Simulation instruction	7	8, 14, 42, 53, 55, 57, 63
Small group instruction	7	8, 41, 46, 50, 53, 56, 57
Time management	2	8, 46

**Table 4:** Summary of educational strategies in resident-as-teacher curricula.

Educational Strategy	Number of Papers	References
Didactic lectures	22	5, 7, 8, 12, 17, 29, 33, 37, 38, 42, 47, 57, 58, 64, 71, 75, 86, 87, 93, 95, 103, 104
Direct observation and feedback	13	29, 31, 49, 56, 57, 59, 66, 69, 71, 87, 88, 94, 104
Iterative reminders / staged repetition	4	29, 31, 61, 71
Simulation/role playing	12	12, 14, 31, 37, 57, 64, 69, 70, 75, 87, 88, 91
Small groups	6	12, 36, 37, 56, 69, 93
Virtual sessions/electronic handouts	7	5, 41, 43, 61, 71, 86, 97
Workshops	21	4, 15, 17, 31, 33, 37, 38, 44, 48, 52, 54, 57, 59, 62, 64, 65, 66, 83, 87, 98, 104

**Table 5:** Summary of methods of outcome assessments in resident-as-teacher curricula.

Educational Strategy	Number of Papers	References
Observed Structured Teaching Evaluation	12	14, 15, 17, 37, 41, 49, 54, 69, 70, 75, 87, 98
Survey of faculty	4	7, 17, 54, 58
Survey of learners	36	4, 7, 8, 12, 14, 17, 29, 31, 33, 37, 38, 42, 44, 47, 48, 51, 54, 56, 57, 58, 61, 62, 65, 66, 71, 73, 83, 86, 91, 94, 96, 97, 100, 103–105
Semi-structured interview	1	59
