# Supplementary material for: Resident-as-Teacher Curriculum: An Evidence-based Guide to Best Practices from the Council of Residency Directors in Emergency Medicine
Source: West J Emerg Med. 2025 Sep 24;26(5):1135–43. doi: 10.5811/westjem.41493 (PMC12591658; doi:10.5811/westjem.41493)
Supplement: Supplementary file 1 [file wjem-26-1135-s001.docx]

Supplemental Appendix 1. Search Terms

|  |
| --- |
| "residents as teachers".ti,ab. OR "residents-as-teachers".ti,ab. OR "resident as teacher".ti,ab. OR "resident-as-teacher".ti,ab. OR "RaT program".ti,ab. OR "RaT programs".ti,ab. OR "RaT programme".ti,ab. OR "RaT programmes".ti,ab. OR "RaT programmatic".ti,ab. OR "resident as educator".ti,ab. OR "residents as educators".ti,ab. OR "resident-as-educator".ti,ab. OR "residents-as-educators".ti,ab. OR "RaE program".ti,ab. OR "RaE programs".ti,ab. OR "RaE programme".ti,ab. OR "RaE programmes".ti,ab. OR "RaE programmatic".ti,ab. OR "medical education track".ti,ab. OR "medical education tracks".ti,ab. OR "meded track".ti,ab. OR "meded tracks".ti,ab. OR ((resident OR residents) adj3 (teacher OR teachers OR educator OR educators)).ti,ab. |
