# Supplementary material for: Resident-as-Teacher Curriculum: An Evidence-based Guide to Best Practices from the Council of Residency Directors in Emergency Medicine
Source: West J Emerg Med. 2025 Sep 24;26(5):1135–43. doi: 10.5811/westjem.41493 (PMC12591658; doi:10.5811/westjem.41493)
Supplement: Supplementary file 2 [file wjem-26-1135-s002.docx]

Studies from databases/registers **(n = 2894)**

References from other sources **(n =0)**

Citation searching (n = 0)

Grey literature (n = 0)

**Identification**

Studies included in review **(n = 89)**

Studies excluded **(n = 1237)**

Studies not retrieved **(n = 0)**

Studies assessed for eligibility **(n = 249)**

Studies sought for retrieval **(n = 249)**

Studies screened **(n = 1486)**

Studies excluded **(n = 158)**

book (n = 2)

duplicate (n = 4)

Not in English (n = 5)

No full text available (n = 22)

Not about RaT curriculum (n = 72)

Wrong patient population (n = 3)

abstract only, not full manuscript (n = 50)

References removed **(n = 1408)**

Duplicates identified manually (n = 3)

Duplicates identified by Covidence (n = 1405)

Marked as ineligible by automation tools (n = 0)

Other reasons (n = 0)

**Screening**

**Included**
